# Supplementary figures and images for: Metrics of high cofluctuation and entropy to describe control of cardiac function in the stellate ganglion
Source: eLife. 2022 Nov 25;11:e78520. doi: 10.7554/eLife.78520 (PMC9815826; doi:10.7554/eLife.78520)

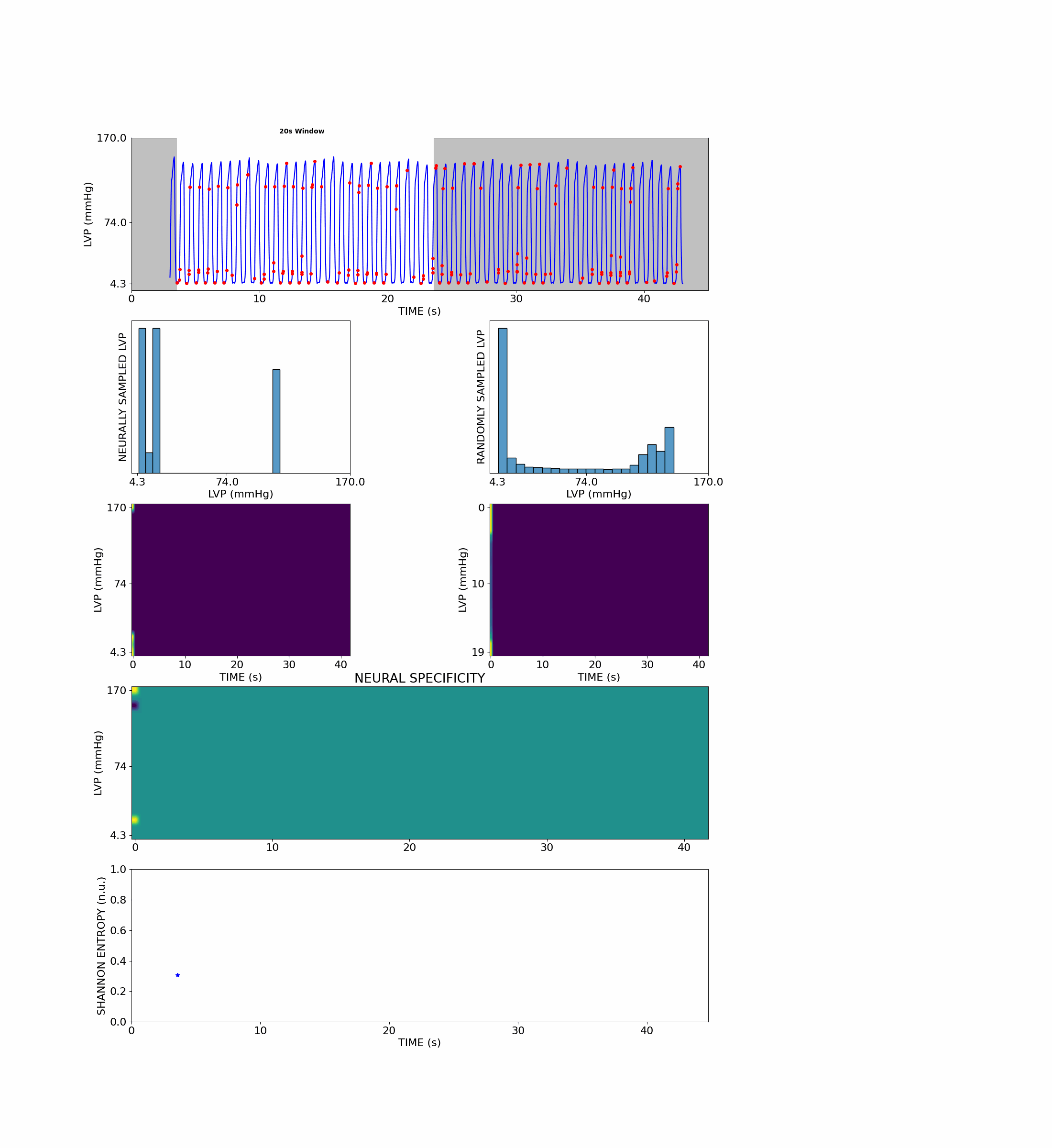

Supplement: Appendix 1—animation 1. — The Animation 1.gif file contains an animation of the building of the neural specificity metricwith respect to left ventricular pressure (LVP). Row 1: Computation of the neural specificity metric for different frames is shown in the form of 20 s moving windows on a 45-s segment of the LVP data. The LVP data are shown in blue tracings with the neural spikes represented as red dots. Row 2: Two normalized histograms for each of the moving windows are calculated and shown in the second row. The histogram of the LVP computed at spike times (neurally sample LVP) is on the left. The histogram of the LVP in the window (randomly sampled LVP) is on the right. Row 3: The computed histograms are then used to compute two matrices in the third row. The matrices contain all the corresponding histograms computed in the previous step arranged vertically with a hard threshold of 0.5 applied, that is, histogram valuesgreater than 0.5 are set to 0.5 (colored yellow). Row 4: The two matrices computed in row 3 are subtracted to obtain the neural specificity metric shown in the fourth row. The color scheme is explained in Appendix 1—figure 4. Row 5: Entropy shown in the fifth row is obtained by calculating the Shannon entropy (such as depicted in Appendix 1—figure 4) for the subtracted histogram in each of the moving windows for the duration of the metric. [file elife-78520-app1-video1.gif]
